# A Distinct Subset of Fibroblastic Stromal Cells Constitutes the Cortex-Medulla Boundary Subcompartment of the Lymph Node

**DOI:** 10.3389/fimmu.2018.02196

**Published:** 2018-10-02

**Authors:** Arata Takeuchi, Madoka Ozawa, Yasuhiro Kanda, Mina Kozai, Izumi Ohigashi, Yoichi Kurosawa, Md Azizur Rahman, Toshihiko Kawamura, Yuto Shichida, Eiji Umemoto, Masayuki Miyasaka, Burkhard Ludewig, Yousuke Takahama, Takashi Nagasawa, Tomoya Katakai

**Affiliations:** ^1^Department of Immunology, Niigata University Graduate School of Medical and Dental Sciences, Niigata, Japan; ^2^Division of Experimental Immunology, Institute of Advanced Medical Sciences, University of Tokushima, Tokushima, Japan; ^3^Department of Immunology, School of Allied Health Sciences, Kitasato University, Sagamihara, Japan; ^4^School of Medicine, Niigata University, Niigata, Japan; ^5^Laboratory of Immune Regulation, Department of Microbiology and Immunology, Osaka University Graduate School of Medicine, Osaka, Japan; ^6^MediCity Research Laboratory, University of Turku, Turku, Finland; ^7^WPI Immunology Frontier Research Center, Osaka University, Suita, Japan; ^8^Interdisciplinary Program for Biomedical Sciences, Institute for Academic Initiatives, Osaka University, Suita, Japan; ^9^Institute of Immunobiology, Kantonal Hospital St. Gallen, St. Gallen, Switzerland; ^10^Experimental Immunology Branch, National Cancer Institute, National Institutes of Health, Bethesda, MD, United States; ^11^Laboratory of Stem Cell Biology and Developmental Immunology, Graduate School of Frontier Biosciences, Osaka University, Suita, Japan

**Keywords:** chemokines, deep cortex, fibroblastic stromal cells, lymph node, medulla, subcompartment

## Abstract

The spatiotemporal regulation of immune responses in the lymph node (LN) depends on its sophisticated tissue architecture, consisting of several subcompartments supported by distinct fibroblastic stromal cells (FSCs). However, the intricate details of stromal structures and associated FSC subsets are not fully understood. Using several gene reporter mice, we sought to discover unrecognized stromal structures and FSCs in the LN. The four previously identified FSC subsets in the cortex are clearly distinguished by the expression pattern of reporters including PDGFRβ, CCL21-ser, and CXCL12. Herein, we identified a unique FSC subset expressing both CCL21-ser and CXCL12 in the deep cortex periphery (DCP) that is characterized by preferential B cell localization. This subset was clearly different from CXCL12^high^LepR^high^ FSCs in the medullary cord, which harbors plasma cells. B cell localization in the DCP was controlled chiefly by CCL21-ser and, to a lesser extent, CXCL12. Moreover, the optimal development of the DCP as well as medulla requires B cells. Together, our findings suggest the presence of a unique microenvironment in the cortex-medulla boundary and offer an advanced view of the multi-layered stromal framework constructed by distinct FSC subsets in the LN.

## Introduction

Lymph nodes (LNs) are key sites for the induction of adoptive immunity, and this occurs through the filtering of lymph fluid that is drained form peripheral tissues and the monitoring of antigenic information. The LN has a sophisticated tissue architecture that is suitable for immunological functions; it consists of several substructures, in which different sets of immune cells are localized to form functionally distinct areas (1, 2). Such tissue arrangement is probably optimized for the spatiotemporal regulation of immune responses, although the fundamental principles associated with the establishment of this architecture remain largely unclear. In large mammals including humans, two major tissue parts of the LN are evident, namely, the cortex, characterized by dense lymphocyte accumulation in the outer layer, and the medulla, in which blood vessels and lymphatic sinuses are concentrated, in contrast to the relatively sparse arrangement of lymphocytes in the organ center and hilum (1). In the cortex, B cells densely pack to form the follicles (B cell area) at the superficial part, whereas the majority of T cells are localized to the deeper paracortex (deep cortex or T cell area). Upon T cell-dependent responses, activated follicular B cells construct the germinal centers (GCs), which are further composed of two areas, the dark, and light zones. In mice maintained under specific pathogen-free conditions, LNs are relatively small and show a regular architectural pattern with clear tissue polarity; the cortex resides at the hemisphere of the afferent side of lymphatic connections, whereas the medulla is at the opposite side, from which efferent lymph drains. A structural unit composed of one paracortex and several follicles is termed a “compartment” (3) or “lobule” (4, 5), and a single mouse LN has one or more compartments (6). Accordingly, substructures such as the follicle or the paracortex within a compartment should be classified as “subcompartments.”

Previous studies have found that different types of stromal cells of mesenchymal origin, i.e., fibroblastic stromal cells (FSCs), support distinct subcompartments, functioning as a structural backbone and promoting the localization of specific immune cell subsets by producing specific chemoattractants (7–9). The maintenance of a subcompartment likely requires continuous interaction between immune cells and FSCs (10). Follicular dendritic cells (FDCs) residing in the follicular center (or GC light zone) produce the chemokine CXCL13, which attracts B cells and follicular helper T cells expressing CXCR5, and supports their motility within the follicle (11, 12), whereas the development of functional FDCs requires factors produced by B cells (13, 14). In the paracortex, T zone reticular cells (TRCs; also known as fibroblastic reticular cells, FRCs) that produce CCL19 and CCL21-ser (*Ccl21a* gene product) support the localization of T cells and dendritic cells (DCs) expressing CCR7 (15, 16). Marginal reticular cells (MRCs) present in the follicular margin underneath the subcapsular sinus (SCS) also express CXCL13 and are implicated in the delivery of lymph-borne antigens (17, 18). MRCs have been recently shown to be precursors of FDCs (19). A stromal cell subset, CXCL12-expressing reticular cells (CRCs), is localized to the paracortical side of the follicles and upon GC formation, provides functional support for the dark zone (20, 21). Most recently, Cyster and colleagues showed further heterogeneity in FSCs through single-cell RNA sequencing analysis (22), although the functional significance of such highly diversified FSCs remains obscure.

The anatomical region ranging from the deep cortex to the medulla of the LN is presumably important for innate and adaptive responses given the localization of a variety of immune cells including macrophages, NK cells, and plasma cells (23–27). However, knowledge of this area is limited; the indistinct distribution of immune cells, as compared to the cortex, and the intricate structure of intertwined blood vessels and lymphatic sinuses could have hampered in-depth studies. The characteristic anatomies in this area suggest the presence of functionally distinct stromal cells. In this study, we sought to clarify the relevance of FSCs for the arrangement of LN subcompartments by utilizing several gene reporters expressed in stromal compartments. This led to the discovery of a novel FSC type that supports an area in the deep cortex, which was distinct from FSCs in the T cell area as well as the medulla. These observations bring about a comprehensive view of multi-layered subcompartments and associated FSC subsets in the LN.

## Materials and methods

### Mice

C57BL/6JJcl and BALB/cAJcl-*nu/nu* mice were purchased from CLEA, Japan. B6.129P2-*Cxcl12*^*tm*2*Tng*^ (*Cxcl12*-EGFP, *Cxcl12*^+/−^), *Ccl21a*-tdTomato (*Ccl21a*^+/−^), *Tg(Ccl19-cre)*^489*Biat*^/B6.129X1-*Gt(ROSA)26Sor*^*tm*1(*EYFP*)*Cos*^/J (*Ccl19*-cre/*R26*-EYFP), and B6.129S2-*Ighm*^*tm*1*Cgn*^/J (μMT) mice were described previously (28–31). B6.129P2-*Cxcl12*^*tm*2*Tng*^ mouse strain (RBRC04200) was provided by the RIKEN BRC through the National Bio-Resource Project of the MEXT, Japan. Mice were maintained and crossed under specific pathogen-free conditions in the animal facility of Niigata University. All animal procedures were approved by the Committee on Animal Research at Niigata University.

### Generation of *Pdgfrb* reporter mice

Genomic fragments of the *Pdgfrb* gene locus were amplified from RENKA ES cell genomic DNA by PCR. The targeting vector was constructed as follows: the second exon of *Pdgfrb* was inserted with an in-frame start codon followed by the gene encoding EYFP (venus), an internal ribosomal entry site (IRES), the gene encoding CreER^T2^, and in reverse orientation, a FRT-flanked neomycin resistance gene (neo^r^) cassette. The linearized targeting construct was electroporated into RENKA B6 mouse ES cells and G418 resistant colonies were screened by Southern blotting using AflII- or HindIII-digested genomic DNA using a neo^r^-flanking probe. Targeted ES clones were injected into B6 blastocysts and chimeras were mated to B6 mice. Targeted alleles were screened by PCR using the primers: 5′-CTTGTCTGGTCTGCATTTCTTGGC-3′ (sense; PDGFRβ-gF); 5′-TGAACTTGTGGCCGTTTACGTCG-3′ (antisense; EGFP-R10).

### Antibodies

The following fluorochrome-conjugated, biotin-conjugated, or unconjugated primary antibodies were purchased: anti-CD3e (145-2C11), anti-B220 (RA3-6B2), anti-CD11c (N418), anti-F4/80 (BM8), anti-CD45 (30-F11), anti-CD31 (390), and anti-podoplanin (8.1.1) (eBioscience); anti-desmin (Abcam); ER-TR7 (BMA); anti-CD35 (8C12), anti-IgD^b^ (217-170), and anti-CD138 (281-2) (BD Biosciences); anti-VCAM-1 (BAF643), anti-RANKL (BAF462), anti-CXCL13 (BAF470), anti-LYVE-1 (BAF2125), anti-LepR (BAF497) (R&D Systems); anti-laminin (LSL); anti-GFP and anti-RFP (MBL). For secondary reagents, PE-, APC-, AlexaFluor488-, 546-, 555-, 594-, or 633-conjugated streptavidin, anti-rabbit IgG, and anti-rat IgG were purchased from Molecular Probes.

### Flow cytometry

Single-cell suspensions were prepared from superficial LNs (cervical, axillary, brachial, inguinal, and popliteal) through digestion with 1 mg/mL collagenase D and 0.1 mg/mL DNase I (Roche Diagnostics) as described (32), and stained with anti-CD45, anti-CD31, and anti-gp38/podoplanin antibodies and propidium iodide. Data were acquired using a FACSCalibur (BD Biosciences) flow cytometer and analyzed with CellQuest (BD Biosciences) or FlowJo.

### Immunohistochemistry

Isolated LNs (inguinal, brachial, cervical, and popliteal) were fixed with 0.05% phosphate buffer containing 0.075 M L-lysine (pH 7.4), 0.01 M NaIO_4_, and 1% paraformaldehyde (PLP fixative) at 4°C for 16–24 h. After fixation, LNs were equilibrated gradually with 10, 20, and 30% sucrose in PBS at 4°C, embedded in OTC compound (Sakura Finetechnical), and frozen at −80°C. Frozen sections (10 μm) were made using a cryostat (Leica Biosystems) and post-fixed with cold acetone for 3 min. To properly evaluate the pattern of subcompartments and the location of FSC subsets, we made LN sections that incorporated the cortex–medulla axis. Sections were stained with antibodies and mounted with Permafluor mountant (Thermo Fisher Scientific). The specimens were examined using an LSM710 confocal microscope (Carl Zeiss) and a FV1200 confocal microscope (Olympus). Digital images were prepared using ZEN (Carl Zeiss), FV10-ASW (Olympus), and Adobe Photoshop CS6 (Adobe Systems).

From image data, the longitudinal fluorescent intensity profile and mean fluorescent intensity of the region of interest (ROI) were measured with ImagePro Plus (MediaCybernetics). Fluorescent density was determined by dividing the mean fluorescent intensity by the ROI area. Percent area of the DCP or medulla was determined by dividing the ROI area by the whole LN area and multiplying by 100. Graphs were made in Microsoft Excel or GraphPad Prism 6.

### Live imaging of B cell migration

B cells were isolated from the LNs and spleen by magnetic cell sorting using a B-cell isolation kit (Miltenyi Biotec) and labeled with 5 μM CMTMR (Invitrogen) at 37°C for 20 min. B cells (1 × 10^7^) were intravenously injected into B6 or *Cxcl12*-EGFP mice, and sacrificed for imaging analysis at 24 h after transfer.

Live imaging of LN explants was performed as described previously (33). Skin-draining LNs isolated from mice were glued onto plastic cover slips (Fisher Scientific) using Vetbond (3M) with the medullary side facing upward. Alternatively, to make tissue slices, LNs were cut with a vibratome (VT1200, Leica) at room temperature. The LNs were placed in a heated chamber (RC-26G, Warner Instruments) and continuously perfused with RPMI1640 medium equilibrated with 95% O_2_/5% CO_2_ at 36.0–36.5°C. Time-lapse images were acquired using a two-photon laser-scanning microscope (LSM710-NLO, Carl Zeiss). The Ti:sapphire laser (Chameleon, Coherent) was tuned to 850 or 880 nm. Stacks of 17–25 x–y optical sections (256 × 256 or 512 × 512 pixels) with 1–3 μm z-spacing were acquired every 20 s for 20–30 min, using emission wavelengths of 495–540 nm (for EGFP) and 575–630 nm (for CMTMR). To obtain static images at higher resolution, 1,024 × 1,024 pixels and 1-μm z-spacing were used for data acquisition. Image stacks were transformed into volume-rendered four-dimensional movies.

Fluorescent objects in images were detected using Imaris software (Bitplane). Cell motility was analyzed by semi-automated tracking of cell centroids; cellular motility parameters were calculated from the x, y, and z coordinates using Microsoft Excel. B cells in contact with FSCs were counted in every time frame of three-dimensional images. Graphs were made in Microsoft Excel or GraphPad Prism 6.

### Bone marrow chimera

Bone marrow chimeric mice were generated as described (34). Briefly, μMT or *Cxcl12*-EGFP/μMT recipient mice were intraperitoneally injected with busulfan (Tokyo Chemical Industry, 30 μg/g body weight) for three times at 7, 5, and 3 days prior to receiving 5 × 10^6^ bone marrow cells from C57BL/6J wild type mice. Two months after the reconstitution, chimeric mice were used for analysis.

### Statistical analysis

GraphPad Prism 6 was used for statistical analyses. The means of two groups were compared by performing an unpaired Student's *t*-test. The Mann–Whitney *U*-test was used to compare nonparametric datasets. *P* values of < 0.05 were considered statistically significant.

## Results

### Differentially expressed gene reporters distinguish the heterogeneity of FSCs in the LN

To determine the precise localization of FSC subpopulations and complicated cellular morphology of the stromal network, we utilized some reporter mice that express fluorescent protein (FP) under the control of genes expressed in LN FSCs. We first generated a knock-in mouse by inserting an EYFP into the *Pdgfrb* gene (*Pdgfrb*-EYFP), a typical marker of mesenchymal cell lineages. We also employed two established reporters, *Cxcl12*-EGFP (28) and *Ccl21a*-tdTomato (31), both of which are knock-in mice strains generated by inserting an FP reporter in chemokine genes. In addition, we included *Ccl19*-cre BAC transgenic mice intercrossed with the *Rosa26*-stop^flox^-EYFP allele (*Ccl19*-cre/*R26*-EYFP) (29), in which cells that express or have once expressed CCL19 are marked with EYFP expression.

**Figure 1:**
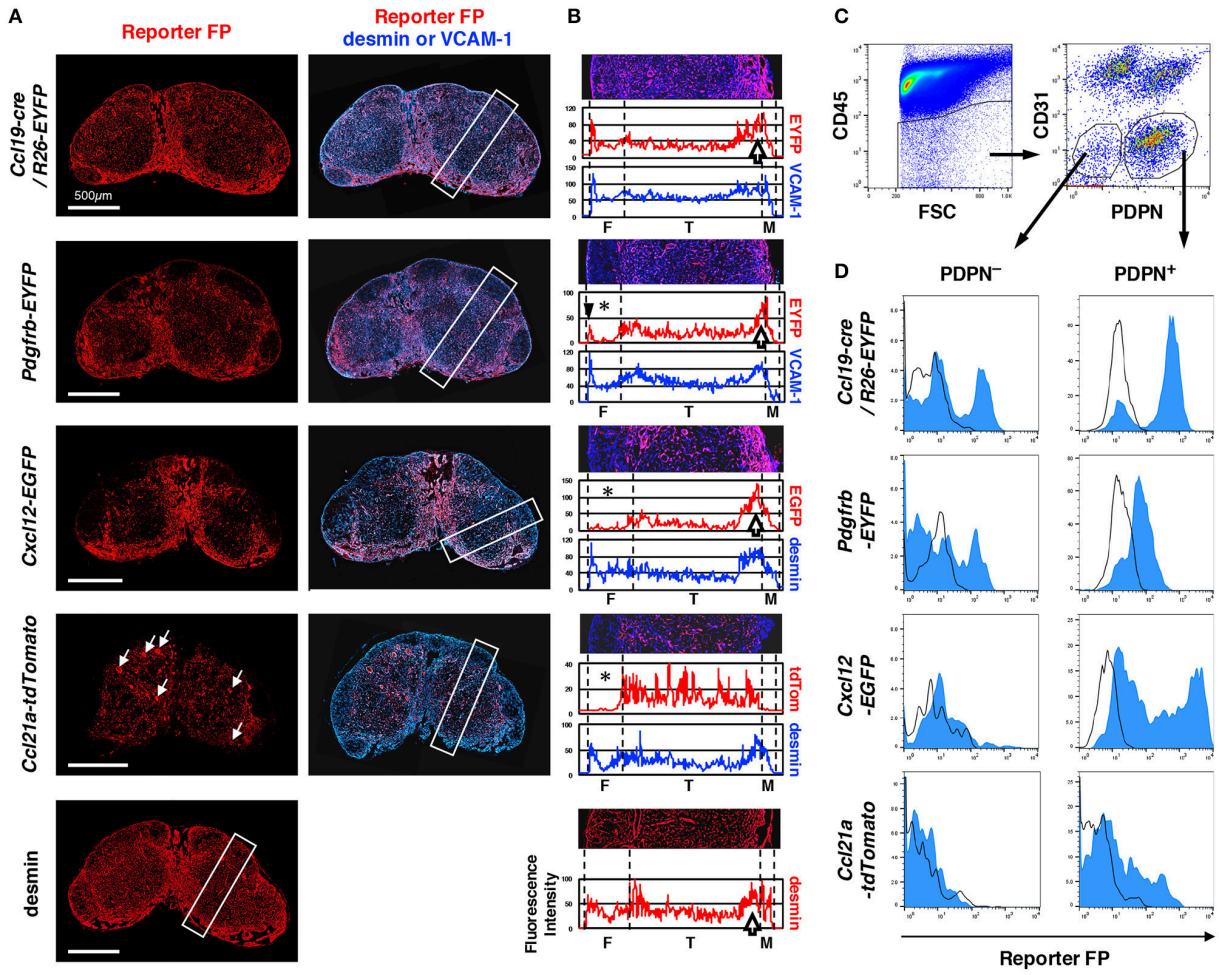
Differences in LN stromal structural patterns based on gene reporters. **(A)** Whole views of inguinal LNs isolated from various reporter mice. Vertical sections of LNs were stained with antibodies against fluorescent protein (FP) and desmin or VCAM-1. White arrows in the panel of *Ccl21a*-tdTomato indicate HEVs. **(B)** Average fluorescent intensity profiles of the rectangular boxed region along with the cortex-medulla axis in **(A)**. The image of the region of interest (ROI) is replicated in the upper part and the boundary between subcompartments is indicated by dotted lines. Note the absence of or low FP expression in the follicular area (asterisks), the sharp peak in FP in the outer margin of the follicle (arrowhead in *Pdgfrb*-EYFP), and the higher signal around the boundary of the T cell area and medulla (open arrows). F, follicle; T, T cell area (paracortex); and M, medulla. **(C)** Flow cytometric analysis of FSCs. CD45^−^ LN cells (left) were further fractionated by CD31 and PDPN expression (right), and the CD31^−^ non-endothelial fraction was defined as FSCs including PDPN^+^ conventional fibroblastic reticular cells (FRCs) and PDPN^−^ cells (gated, respectively). **(D)** FP expression in PDPN^+^ and PDPN^−^ cells. Open histograms show the corresponding cell fraction of wild type mice.

LNs from each reporter mouse showed significant differences in the pattern of FP expression (Figure 1A). The average fluorescence intensity profile of the short side of the rectangular region set along the cortex–medulla axis in images represented the characteristics of the patterns (Figure 1B). Desmin is a suitable marker for the identification of mesenchymal cells in LN sections (8). We confirmed that an anti-desmin antibody clearly stains FSCs in all areas of the LN (data not shown); thus, we used this as a standard marker of FSCs. We also utilized VCAM-1 as another FSC marker, which is suitable for examining the whole LN structure. In *Ccl19*-cre/*R26*-EYFP mice, FP signal was detected in FSCs of all areas, similar to that observed for desmin (Figures 1A,B), suggesting that most FSC types (or subsets) once expressed cre recombinase under the control of the *Ccl19* transgenic locus (29). The *Pdgfrb*-induced FP was also widely observed in the LN. However, a significant feature of the PDGFRβ reporter was the absence of signals in the follicular areas, whereas relatively high and dense reporter expression was observed around the boundary of the cortex and medulla. Desmin showed a similar high density in the cortex-medulla (C-M) boundary. The expression of the CXCL12 reporter was relatively low in the follicles, but was significantly high at the C-M boundary. In sharp contrast, CCL21-ser expression was most evident in paracortical FSCs as expected, in addition to high endothelial venules (HEVs) (Figure 1A, white arrows). Therefore, these reporters could provide detailed information regarding specific stromal structures in subcompartments constructed by FSCs.

To determine the proportion of FSCs expressing reporter FPs, we analyzed cells from collagenase-digested LNs by flow cytometry. The CD45^−^CD31^−^ fraction includes non-hematopoietic and non-endothelial FSCs, and in this population, podoplanin (PDPN)^+^ cells have been regarded as FRCs (16) (Figure 1C). Our observations clearly showed that the “FRC fraction” is composed of heterogeneous cells displaying varying degrees of FP expression (Figure 1D). In LNs from *Ccl19*-cre/*R26*-EYFP and *Pdgfrb*-EYFP mice, the majority of cells in the FRC fraction exhibited reporter expression, although the FP signal was undetectable in a small fraction of cells. In contrast, a broad expression of reporter FP expression was detected in the FRC fraction of *Cxcl12*-EGFP mice, whereas only a limited proportion of cells expressed the FP reporter in *Ccl21a*-tdTomato mice. These results indicate that the conventional CD45^−^CD31^−^PDPN^+^ FRC fraction, based on flow cytometric analysis, is a mixture of FSC subsets indicated by the reporters.

### Cortical FSC subsets can be distinguished based on the expression pattern of reporters

**Figure 2:**
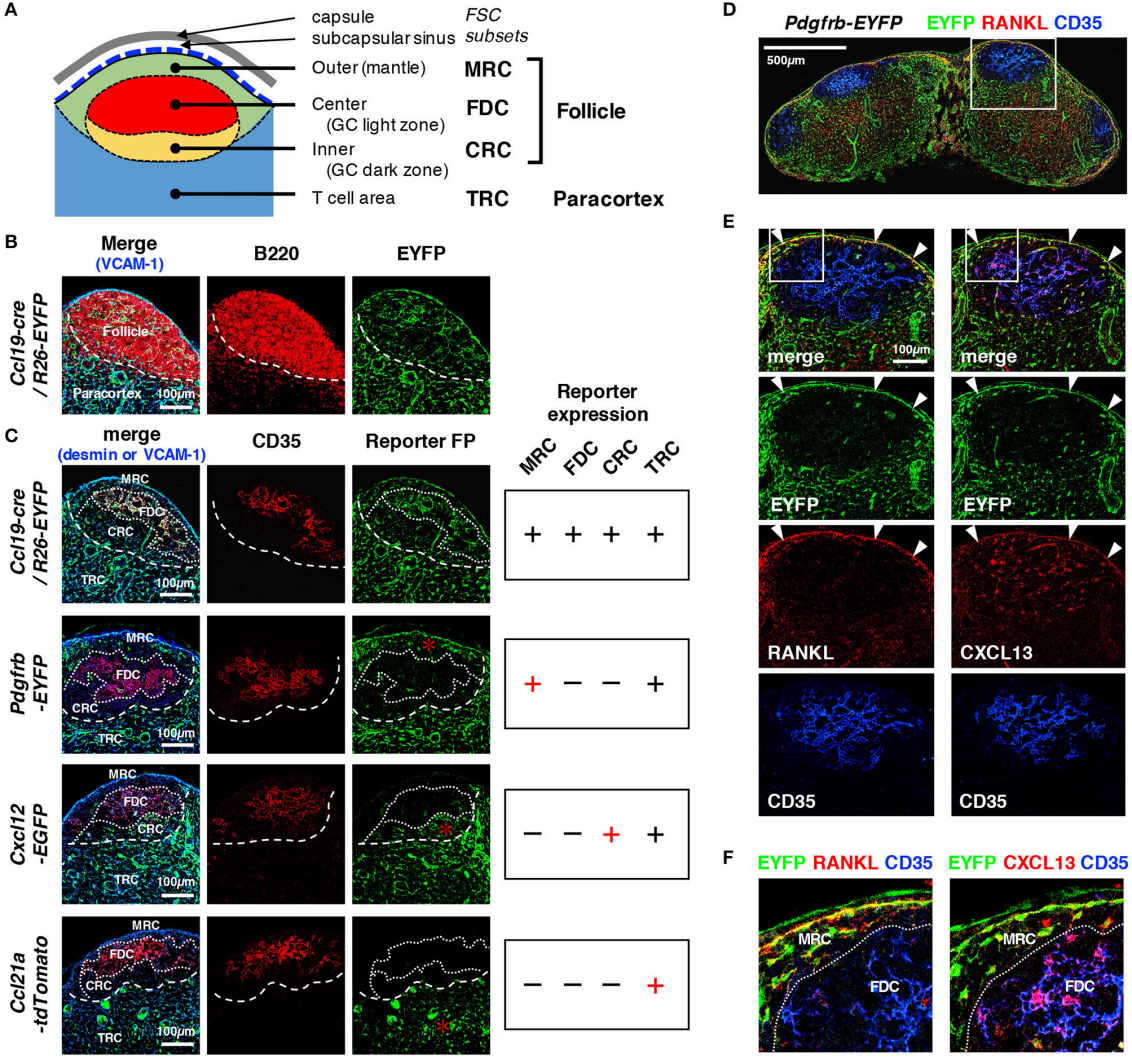
Distinct patterns of reporter expression in cortical FSC subsets in mouse LNs. **(A)** Schematic representation of subcompartments and associated FSC subsets in the outer cortex. **(B)** Close-up view of the outer cortex. The border of the follicle and paracortex is indicated by the localization of B cells (dotted line). **(C)** The patterns of FP expression in reporter mice. CD35 expression is an indicator of the FDC network in the follicular center. Territorialities of the network of FSC subsets are indicated by dotted lines. FP expression in each FSC subset is summarized on the right. Asterisks indicate characteristic FP expression. **(D–F)** Expression of RANKL and CXCL13. Sections of an inguinal LN from *Pdgfrb*-EYFP mouse were stained for the indicated markers. The boxed regions in **(D,E)** are magnified in **(E,F)**, respectively. Note that MRCs express both RANKL and CXCL13 (arrowheads in **E**), whereas FDCs express CXCL13 but not RANKL.

We next compared reporter expression in known cortical FSC subsets. Within follicles that are characterized by B cell localization to the outer cortex, FDCs expressing CD35 were present in the follicular center, whereas MRCs and CRCs were localized to the SCS- and paracortical-side of the follicle, respectively (Figures 2A–C). Outside the follicles, a network of TRCs was found to cover the paracortical T cell area. We found that all four types of FSCs displayed fluorescent signals in the LNs of *Ccl19*-cre*/R26*-EYFP mice (Figures 2B,C). In *Pdgfrb*-EYFP mice, FDCs and CRCs showed virtually no reporter expression, in sharp contrast to MRCs and TRCs, both of which exhibited clear FP signals (Figure 2C). In particular, substantial PDGFRβ reporter expression in MRCs, which was highlighted by their interconnected morphology in the SCS-lining, co-localized with RANKL, and CXCL13, typical markers of MRCs (Figures 2D–F). We detected significant CXCL12 expression in CRCs and TRCs but not in MRCs and FDCs, although the EGFP signal in CRCs was relatively weak compared to that in other parts of the LN (as described below), suggesting moderate CXCL12 expression (Figure 2C). Moreover, CRCs were not often prominent in follicles that did not bear GCs. As expected, TRCs were the only cell type that expressed CCL21-ser among these cortical subsets (Figure 2C). Collectively, cortical FSC subsets showed different properties of reporter expression, which clearly distinguishes these subsets from each other.

### A newly defined FSC subset in the deep cortex periphery (DCP)

**Figure 3:**
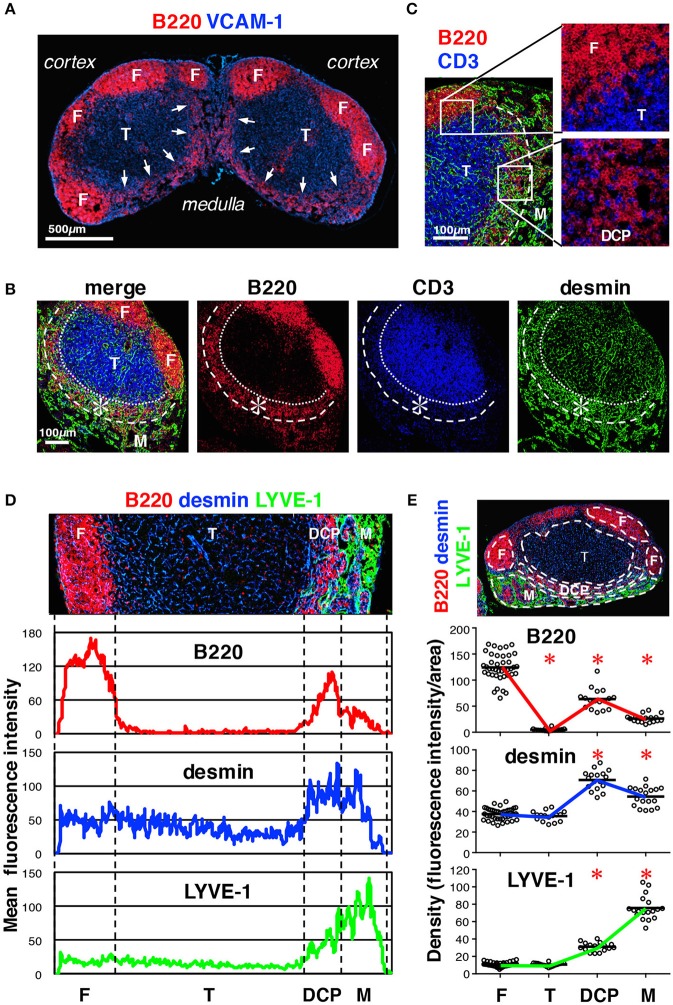
Characterization of the DCP of mouse LNs. **(A)** Substantial B cell accumulation in the periphery of the deep cortex adjoining the medulla (inguinal LN, arrows). **(B)** The DCP is a belt-like zone surrounding the bottom of the T cell area (asterisks). **(C)** B and T cells were intermingled in the DCP (higher magnification view in the lower right), as compared to the superficial cortex, in which they were clearly segregated (upper right). Laminin signals are eliminated in the right micrographs. **(D)** Enrichment of desmin correlated with B cell accumulation in the DCP. Average fluorescent intensity profiles of B220, desmin, and LYVE-1 along the cortex–medulla axis. Desmin signals were also high in the medulla. **(E)** Quantitative analysis of signal densities in LN subcompartments. The micrograph is representative of the region of interest (ROI) from the image data. Marker densities in each region are plotted (open circles) in the lower graphs (bars, mean; asterisks, significantly different compared to the follicles, *p* < 0.0001).

To characterize the stromal structures in the deep cortex and medulla, we examined these regions in more detail and noticed an area in which B cells showed belt-like accumulation near the C-M boundary; this comprised the bottom of a bowl-shaped region of the T cell area (Figures 3A,B). We reasoned that this structure corresponds to the deep cortex periphery (DCP), originally reported by Sainte-Marie et al. which is a region with dense reticular meshwork near the C-M boundary (3, 35). Despite low B cell density, compared to that in the follicles, the DCP seemed unique in terms of immune cell localization, in that B cells and T cells were intermingled (Figure 3C). Moreover, the DCP corresponded to the region of high desmin density (Figures 3D,E) and high reporter expression in *Pdgfrb*-EYFP and *Ccl19*-cre/*R26*-EYFP mice (Figures 1A,B arrows), suggesting that FSCs are particularly concentrated in this area. The boundary of the DCP and medulla was occasionally indistinct, since the medullary cords and/or the lymphatic sinuses invaded into the DCP. However, based on the structural continuity with the cortex, namely lymphocyte density and the connection with the stromal network, we concluded that the DCP comprises the part of the cortex adjacent to the medulla.

**Figure 4:**
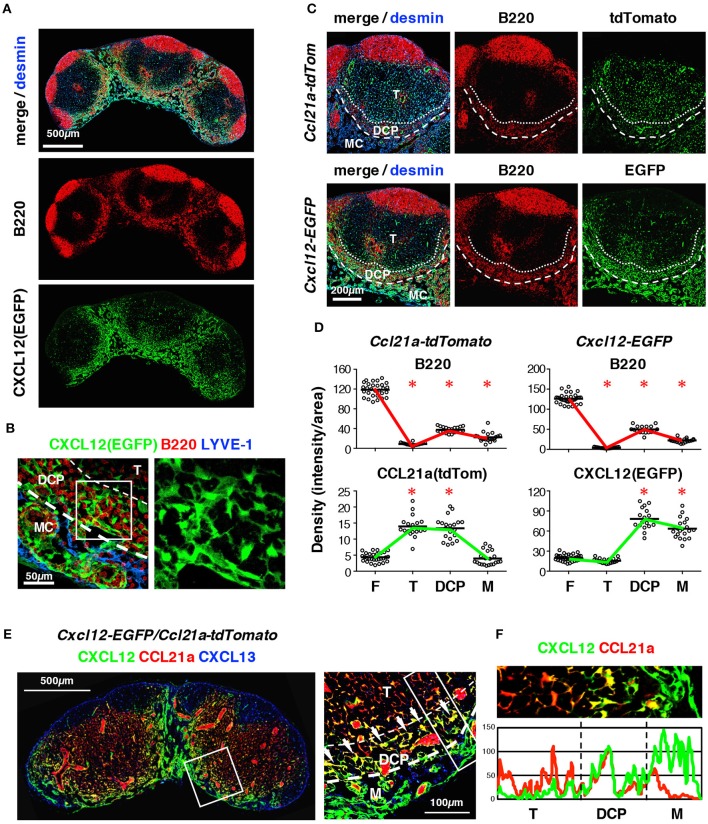
The DRC comprises a unique FSC subset that expresses CXCL12 and CCL21-ser in the DCP of mouse LNs. **(A)** Enrichment of CXCL12^high^ FSCs in the DCP and medulla. Section of a cervical LN from a *Cxcl12*-EGFP mouse stained for B220 and desmin. **(B)** Z-projection image (0.5 μm interval × 10) of the DCP and medulla. Boxed area is magnified in the right panel (EGFP only), showing the dense network of DRCs. **(C)** Comparative views of FP expression in the DCP between *Ccl21a*-tdTomato and *Cxcl12*-EGFP mouse strains. **(D)** Quantitative analysis of signal densities in LN subcompartments in *Ccl21a*-tdTomato and *Cxcl12*-EGFP mice (bars, mean; asterisks, significantly different compared to the follicles, *p* < 0.0001). **(E)** Inguinal LN section from *Cxcl12*-EGFP/*Ccl21a*-tdTomato mouse with staining for CXCL13. The boxed area in left micrograph is magnified on the right. Bright yellow signals (arrows) indicate the cells highly expressing both CXCL12 and CCL21-ser. **(F)** Average fluorescent intensity profiles of EGFP (CXCL12) and tdTomato (CCL21-ser) in the boxed area in **(E)** right.

**Figure 5:**
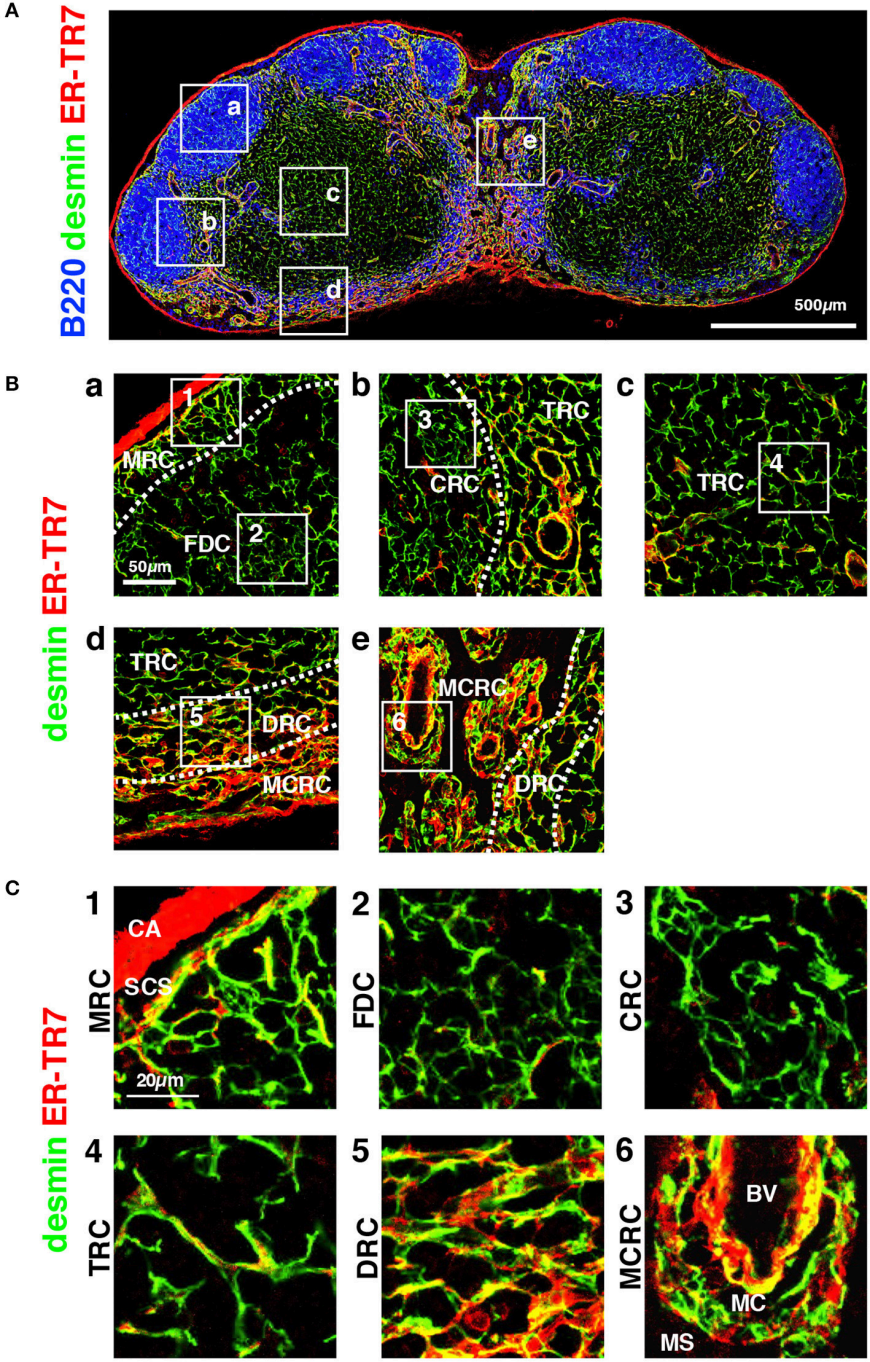
Morphology and network structures of FSC subsets. **(A)** Whole view of an inguinal LN section with staining for B220, desmin, and ER-TR7. **(B)** Highly magnified z-projection images (1 μm interval × 5) of the boxed areas (a–e) in **(A)**. **(C)** Higher magnification views of the boxed areas (1–7) in **(B)**. BV, blood vessel; CA, capsule; CRC, CXCL12-expressing reticular cell; DRC, DPC reticular cell; FDC, follicular dendritic cell; MCRC, medullary cord reticular cell; MRC, marginal reticular cell; SCS, subcapsular sinus; TRC, T zone reticular cell.

Importantly, B cell localization in the DCP correlated well with a subpopulation of FSCs that expressed high levels of the CXCL12 reporter (Figures 4A,B); however, small numbers of B cells and CXCL12^high^ cells were also present in the medulla. FSCs in the DCP substantially expressed CCL21-ser as well (Figures 4C,D). Mice bearing *Cxcl12*-EGFP/*Ccl21a*-tdTomato double reporter demonstrated that each FRC in the DCP area highly expresses both CXCL12 and CCL21a, in comparison with FSCs in the T cell area and the medulla (Figures 4E,F). In addition, they were morphologically distinct from TRCs based on the fact that they had more connections with neighboring cells and the dense network tended to be aligned laterally along the B-cell belt (Figure 5). Thus, the DCP is a unique subcompartment supported by specialized FSCs expressing both CXCL12 and CCL21-ser. Based on these findings, we concluded that FSCs in the DCP can be regarded as a new subset and called these DCP reticular cells (DRCs).

**Figure 6:**
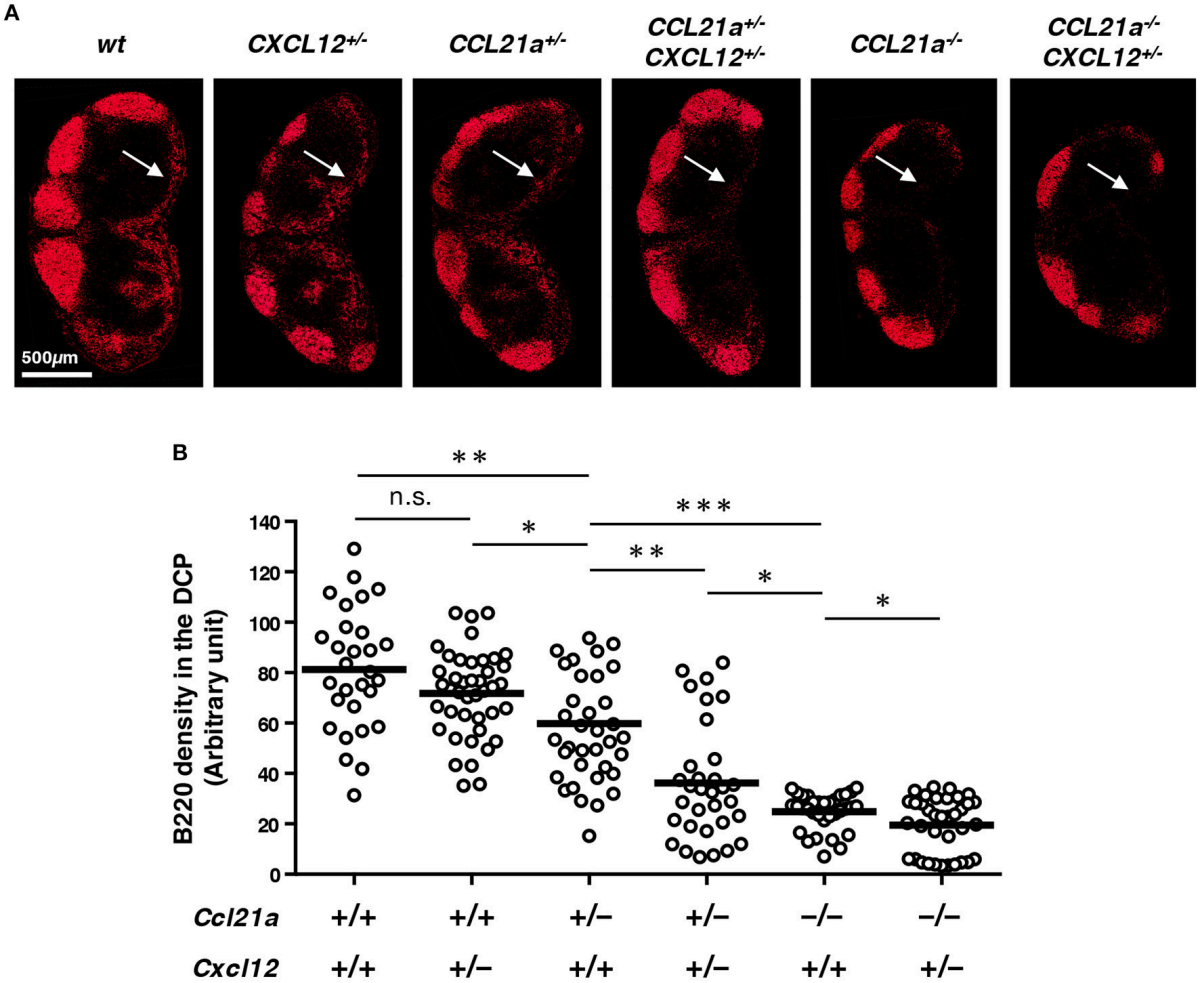
CCL21-ser and CXCL12 synergistically control the localization of B cells in the DCP. **(A)** LN sections from mice with the indicated genotypes were stained for B220. Representative images of inguinal LN are shown. Arrows indicate the DCP. **(B)** Signal densities of B220 in the DCP area of various LNs are plotted (open circles). Bars: mean, n.s., not significant; *p < 0.05, **p < 0.005, ***p < 0.0001.

To determine whether B cell localization in the DCP depends on CXCL12 and CCL21-ser, we next compared various combinations of deficiency in these chemokine genes taking advantage of knock-in alleles (Figure 6). Interestingly, CCL21-ser seemed critical, as the accumulation of B cells in the DCP was significantly reduced in *Ccl21a*^+/−^ (*Ccl21a*-tdTomato) mice compared to that in wild type controls (*Ccl21a*^+/+^*Cxcl12*^+/+^) and was further dramatically decreased in *Ccl21a*^−/−^ mice, in a gene-dosage dependent manner. In contrast, the density of DCP-B cells was slightly but not significantly decreased in *Cxcl12*^+/−^ (*Cxcl12*-EGFP) mice. However, haploinsufficiency of *Cxcl12* gene clearly affected B cell density under *Ccl21a*^+/−^ and *Ccl21a*^−/−^ settings (*Ccl21a*^+/−^*Cxcl12*^+/+^ vs. *Ccl21a*^+/−^*Cxcl12*^+/−^ and *Ccl21a*^−/−^*Cxcl12*^+/+^ vs. *Ccl21a*^−/−^*Cxcl12*^+/−^), suggesting that *Cxcl12* also contributes to the localization of B cells in the DCP. Moreover, DCP-B cells almost disappeared in LNs of *Ccl21a*^−/−^*Cxcl12*^+/−^ mice. Together, these indicate that two chemokines produced by DRCs synergistically control B cell accumulation in this area.

### B cell migration in the DRC network

**Figure 7:**
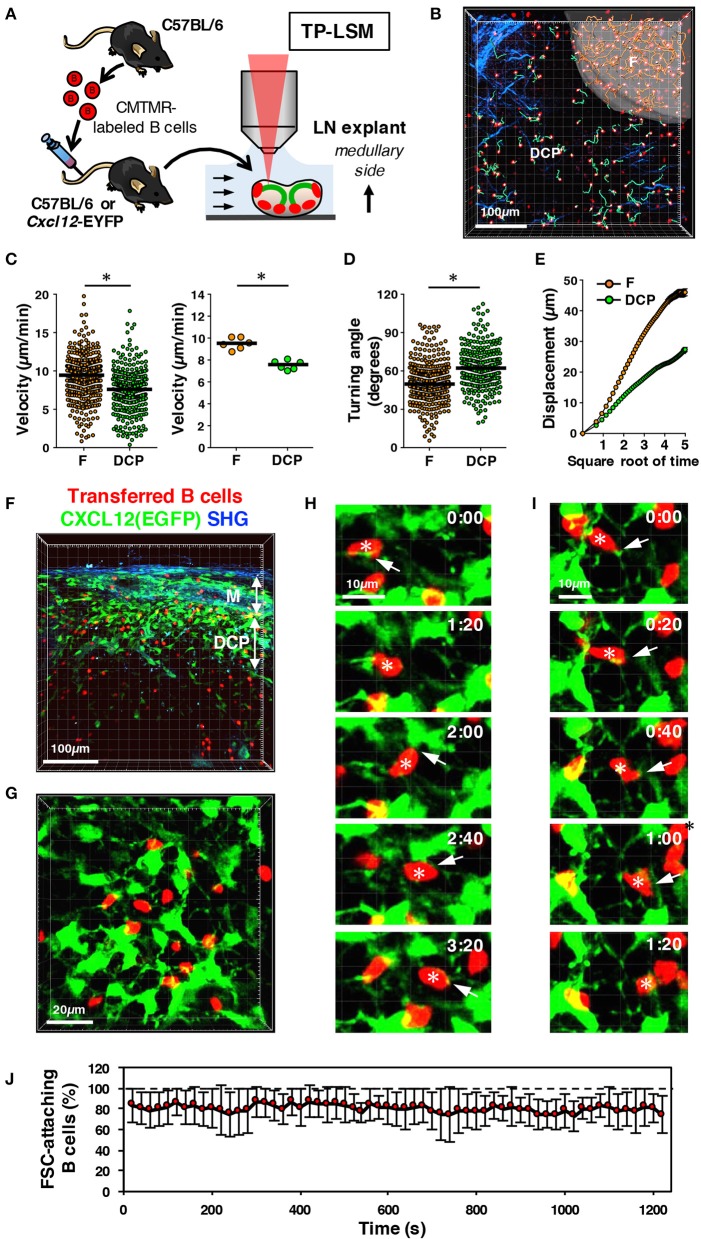
Migration ability of DCP B cells interacting with DRC network in mouse LNs. **(A)** Schematic representation of experimental procedures. B cells isolated from wild type mice were labeled with CMTMR and transferred to recipient B6 or *Cxcl12*-EGFP mice. After 24 h, LN explants were examined with two-photon laser-scanning microscopy (TP-LSM) from the medullary side. **(B)** Three-dimensional reconstruction image (red, B cells; blue, collagen fibers detected by second harmonic generation) with the migration trajectories of B cells in the follicle (F, pseudo-surfaced region; trajectories, orange lines) and extrafollicular area including the DCP and medulla (green lines). **(C)** Migration velocity of B cells in the follicle (F) and DCP. The plot shows the mean velocity of individual cells (circles), as well as the median (horizontal bars). The right plot shows the median velocity for individual experiments (circles) and the mean of six experiments (horizontal bars); *p < 0.0001. **(D)** Mean turning angle of individual cells; *p < 0.0001. **(E)** Mean displacement of cells plotted against the square root of time (s). Error bars: ± SEM. **(F)** Three-dimensional reconstruction image of vertically sliced, fixed LNs from *Cxcl12*-EGFP mice transferred with B cells (red, B cells; green, EGFP; blue, collagen fibers (SHG)). **(G)** Snap shot of live imaging of B cell migration in the DCP area of *Cxcl12*-EGFP LNs. Shown is a three-dimensional reconstruction view. **(H,I)** Time-lapse images of B cells interacting with the DRC network. Note that some B cells (asterisks) migrate in contact with the DRC cell body or the extending thin filaments (arrows). **(J)** Frequency of B cells in contact with FSCs in every frame of time-lapse images. Eight independent image sets were analyzed (20 s intervals for 20 min, 61 frames; total 3,953 cells counted in 488 images; mean ± SD).

Given the co-localization of B cells with DRCs in the DCP, we expected a close interaction between them. Within the follicles, B cells are known to migrate to be in contact with the FDC network (8); therefore, we speculated that DCP B cells migrate in a similar manner to the DRC network. To address this, we performed two-photon microscopy on the LNs of mice transferred with fluorescent-labeled B cells (Figure 7A). Examining LN explants from the medullary side and setting the image field to include both follicle and DCP, we compared B cell migration between the two areas (Figures 7A,B and Video S1). Consequently, although B cells in the DCP showed active migration, the velocity and displacement were significantly lower and the turning angle was higher than those in follicular B cells (Figures 7C–E), suggesting that B cell behavior in the DCP clearly differs from that in the follicle. In the LNs from wild type and *Cxcl12*-EGFP mice, we visualized DRCs that formed a three-dimensional dense network (Figures 5, 7F,G and Video S2). B cells migrated along with the thin filaments of DRCs, continuously making points of contact, and frequently changing direction (Figures 7H–J, Video S3). These results suggest the possibility that slower migration of DCP B cells might be due to dense DRC network with an intimate interaction and supports the idea that the DCP is a subcompartment that is physiologically distinct from other areas of the LN.

### FSCs in the medullary cords are distinct from DRCs

**Figure 8:**
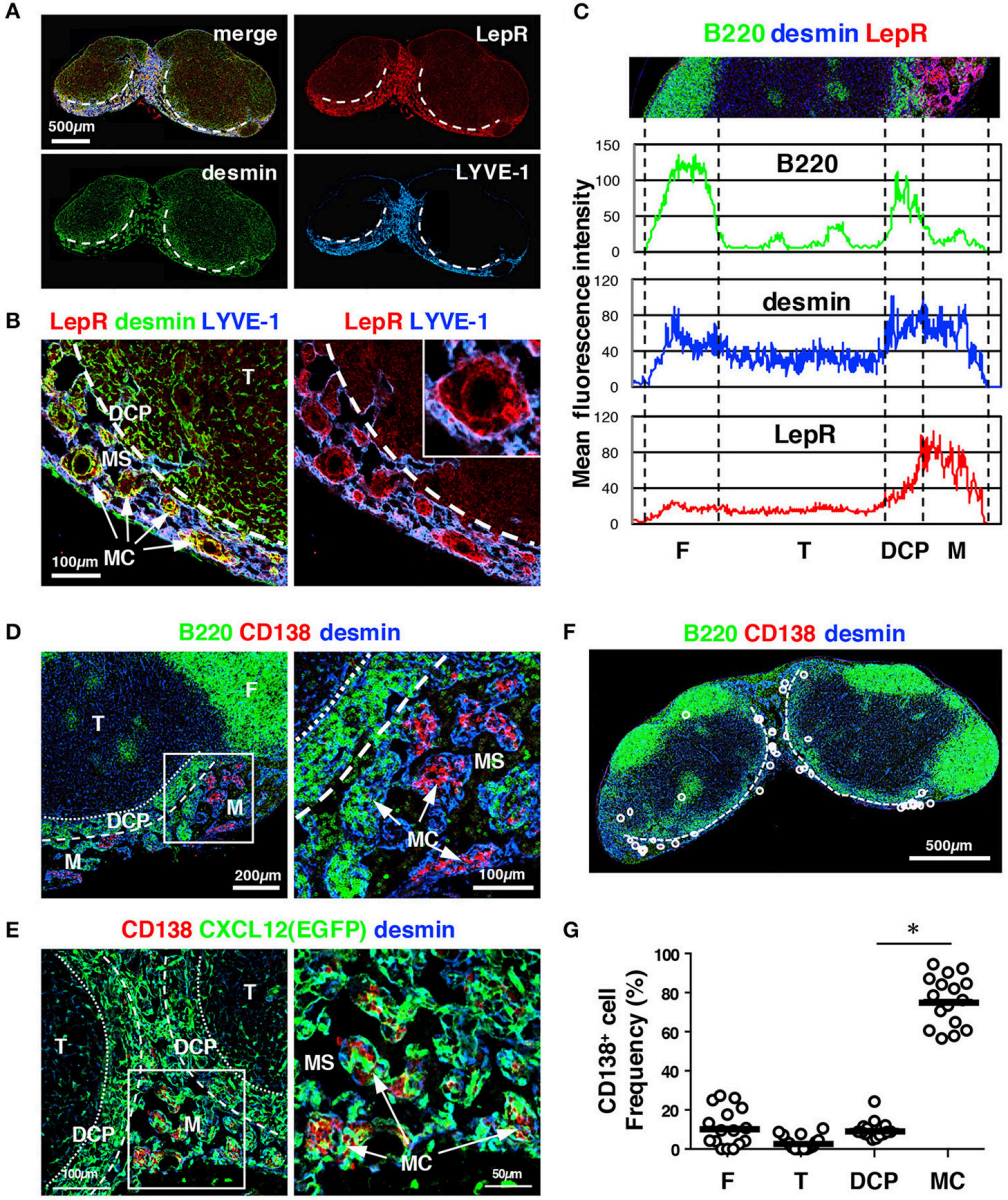
The MC in the mouse LN is a unique structure comprising MCRCs. **(A)** LepR is highly expressed in the medulla. A section of a brachial LN from a wild type B6 mouse was stained with the indicated markers. The dotted lines indicate the boundary between the DCP and medulla. **(B)** FSCs in the MC showed the highest expression of LepR. The inset shows a higher magnification view of a MC. **(C)** Average fluorescent intensity profiles of B220, desmin, and LepR along the cortex–medulla axis. Note that LepR expression is prominent in the medulla, and shows a similar pattern to LYVE-1 in Figure 3D, indicating preferential expression in the medulla. **(D)** Plasma cells preferentially accumulate in the MCs. Shown is a wild type B6 mouse LN. **(E)** Plasma cells make close contacts with MCRCs. Shown is a *Cxcl12*-EGFP mouse LN. **(F,G)** Quantitative analysis of plasma cell localization in LN subcompartments (bars: mean; asterisks: significantly different compared to the follicles, *p* < 0.0001). **(F)** A representative image of plasma cells. The dotted lines indicate the boundary between the DCP and medulla. **(G)** Proportions of plasma cells in each subcompartment compared to the total number in the LN were plotted (open circles; bars: mean; *p < 0.0001).

The medulla is essentially composed of the medullary sinus (MS) and the medullary cords (MCs) (5, 23). As the MS is a continuous luminal structure of lymphatic endothelial cells, FSCs exist primarily in the MC, which is a mesenchymal sheath-like network around blood vessels harboring several types of immune cells. To elucidate the characteristics of FSCs in the MC, we searched for markers that could be detected by antibody staining of tissue sections; the candidate molecules were selected from a list of genes highly expressed in LN stromal cells, as reported previously (32) or based on a public database (ImmGen) (36). Among these candidates, we found that a polyclonal antibody against leptin receptor (LepR) strongly stained the stromal structure of MCs (Figures 8A–C).

Plasma cells are known to be distributed in the MCs, and their migration and localization are mediated in part by CXCL12 (26). We confirmed that CD138^+^ plasma cells accumulated in a fraction of the MCs in unimmunized mice and made close contact with desmin^+^ cells (Figure 8D). FSCs in the MC showed high FP expression in *Cxcl12*-EGFP mice and harbored clusters of plasma cells within the network, whereas plasma cells were rarely observed in the DCP (Figure 8E). Quantitative analysis supported this preferential plasma cell localization in the MC but not in the DCP (Figures 8F,G), whereas a substantial fraction of B cells was also localized to the MC (Figures 3, 4). Together, these results suggest that the microenvironment of the MC is distinguishable from the DCP, which is composed of a different FSC subset. Hence, we defined this subset as medullary cord reticular cells (MCRCs).

### Optimal construction of the medulla and DCP depends on the presence of B cells

**Figure 9:**
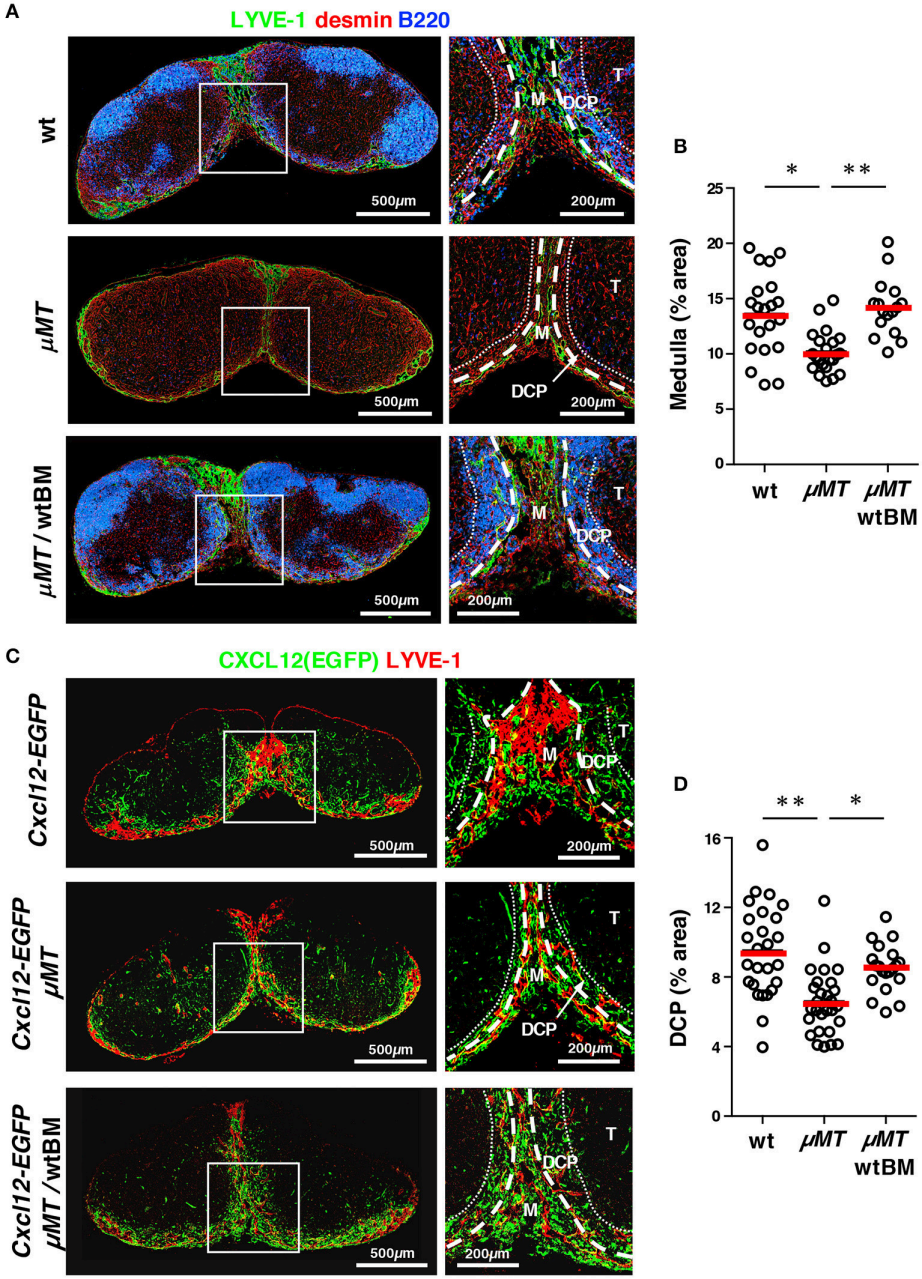
B cells control the optimal development of the medulla and DCP in mouse LNs. **(A)** Development of the medulla was attenuated in the LNs of μMT mice, which was restored with bone marrow transfer. Vertical sections of inguinal LNs isolated from wild type B6, μMT mice, or μMT mice reconstituted with wild type bone marrow (wtBM) were stained with antibodies against LYVE-1, desmin, and B220. Representative images are shown. Higher magnification view of the boxed region is shown in the right panel. **(B)** Proportion of medulla in the LNs (bars: mean; *p < 0.005). **(C)** The DCP was attenuated in *Cxcl12*-EGFP/μMT mice LNs, which was restored with bone marrow transfer. Vertical sections of brachial LNs isolated from *Cxcl12*-EGFP, *Cxcl12*-EGFP/μMT mice, or *Cxcl1*2-EGFP/μMT mice reconstituted with wild type bone marrow (wtBM) were stained with antibodies against LYVE-1, desmin, and B220. Higher magnification view of the boxed region is shown in the right panel. **(D)** Proportion of DCP in the LNs (bars: mean; *p < 0.005, **p < 0.0001).

Given the close association between B cell localization and the DCP and medulla, the integrity of these anatomical structures might be related to B cells. For this, we examined LNs from B cell-deficient μMT mice. Quantitative immunohistochemistry demonstrated that the medulla including the MC and MS was poorly formed in the LNs of these mice compared to that in wild type controls; in addition, expansion or extension of the medullary sinus, stained by LYVE-1, was significantly limited (Figures 9A,B). We also analyzed the DCP area in μMT mice that were crossed with *Cxcl12*-EGFP mice, and were able to identify a DCP-like area with a relatively dense population of FSCs, and with CXCL12 reporter expression even in the absence of B cells. However, we found that this area was clearly attenuated in the μMT background (Figures 9C,D). Reconstitution of B lymphopoiesis in μMT mice with the transfer of wild type bone marrow significantly restored medullary structures in LNs (Figure 9). Therefore, these results suggest that B cells control the optimal development of the DCP as well as the medulla in the LN.

## Discussion

**Figure 10:**
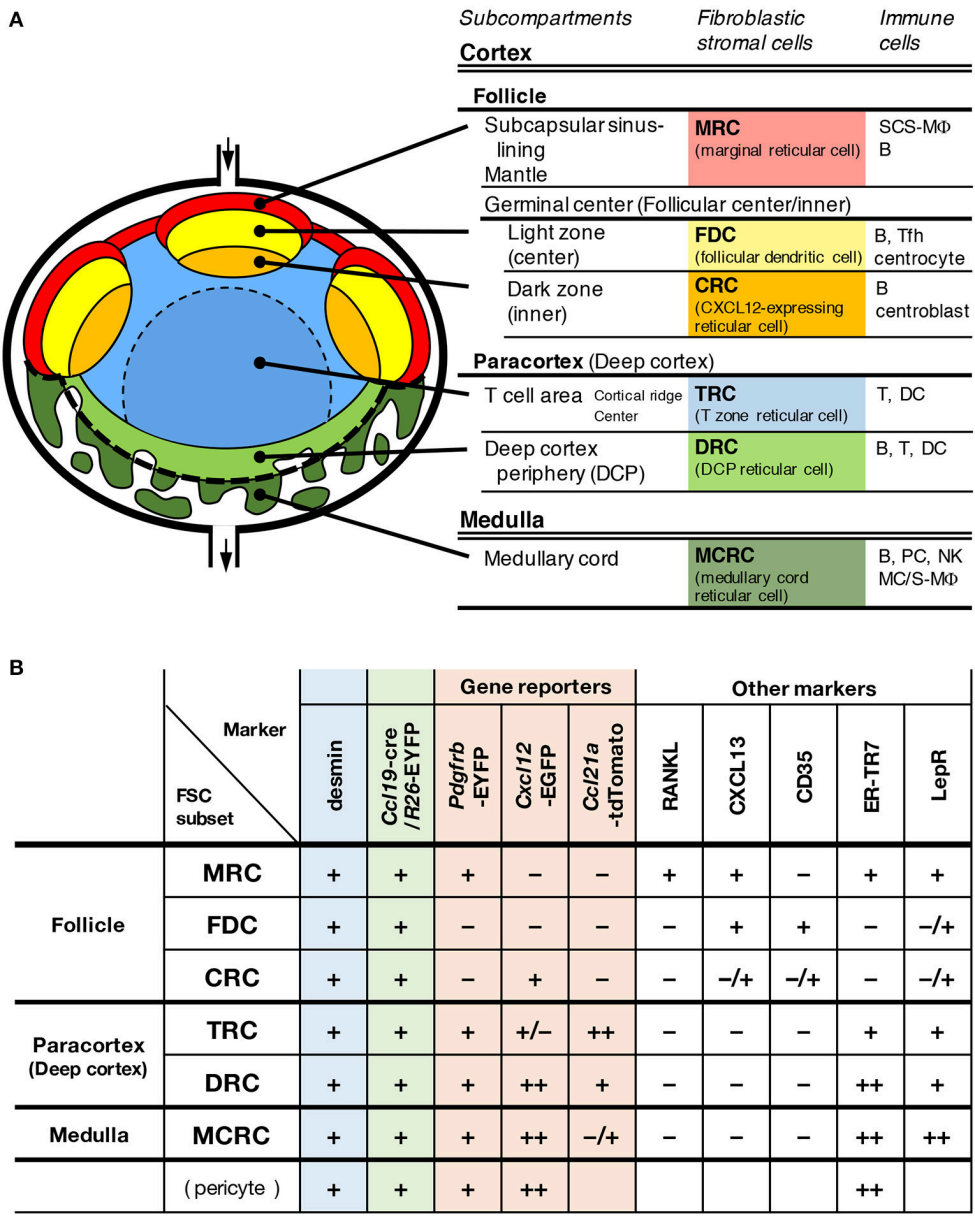
A model of multi-layered subcompartments and associated FSC subsets in mouse LNs. Schematic representation of subcompartmental structures **(A)** and summary of reporter and marker expression in FSC subsets **(B)**.

Tissue infrastructure is likely essential for the spatiotemporal regulation of dynamic immune responses and homeostasis in the LN. This study revealed that a unique FSC subset constitutes the framework of a subcompertment in the deep cortex, which was previously poorly understood. In addition to the newly characterized structure, our observations show that the LN is composed of complex multi-layered subcompartments (Figure 10). The definition of a subcompartment is an anatomical domain with a substantial expanse of space and the localization of a particular set of immune cells within the territorialized network of the FSC subset. From this perspective, the LN has six major subcompartments and at least the same number of corresponding FSC subsets. The differences in the pattern of gene reporters and marker expression among FSCs suggest their distinct roles in tissue organization and immune regulation. In terms of the expression of lymphoid chemokines, the ordered pattern of CXCL13–CCL21-ser–CXCL12 along the cortex–medulla axis is remarkable, and this could be a central determinant of the global LN architecture and immune cell localization. The dense networks of two FSC subsets on the medullary side, both of which highly express CXCL12, were shown to form a bowl-shaped base structure that supports the bottom of the compartment, which appears to be the foundation for entire LN stromal structures.

The boundaries of subcompartments, determined by the localization of motile immune cells, are not necessarily clear and in some cases, FSC subsets were found to be intermingled around the borders. FSCs that did not meet the current criterion might exist locally or be induced in some situations (37). The stromal structure of the paracortical side of the boundary between the follicle and T cell area, called the cortical ridge (CR), is especially complex, and in this region, blood vessels including HEVs, lymphatic sinuses (cortical sinuses), and the reticular network are concentrated to form an intricate anatomy (38). The CR seems to be important for the induction of adoptive immunity, as some DC subsets and activated/memory T cells, as well as innate cells are enriched in this area (24, 39–43). Thus, FSCs in the CR possibly exhibit unique properties; however, there is presently not enough evidence to suggest that they comprise a subset that is distinct from TRCs. Recently, single-cell RNA sequencing analysis of LN stromal cells showed that each subcompartment does not always consist of a uniform FSC but of multiple subpopulations (22). In particular, the CCL21-expressing TRC can be classified into three subsets including Ch25h-expressing CCL19^lo^ TRCs that are observed in the interface between the follicle and T cell area. This powerful approach has the potential to identify a variety of minor populations via subdividing cells based on the overall transcriptional similarities, but might neglect anatomical distributions and differences in the production of a few critical factors. This indicates the importance of both genetic and anatomical approaches to identify FSC subsets.

We obtained clear pictures of stromal structures from the deep cortex to the medulla. To our knowledge, the DCP has never been recognized as a specific region in which B cells are localized. DRCs are a DCP-associated FSC subset that expresses both CXCL12 and CCL21-ser, and forms a dense reticular network. DRC might be included in CCL19^lo^ TRC fraction proposed by Rodda et al. (22). In addition to the substantial expression of *Ccl21*, CCL19^lo^ TRCs showed the highest mean expression of *Cxcl12, Pdgfrb, Tnfsf13b*, and *Il7* among the three TRC subsets. Remarkably, the authors discussed that CCL19^lo^ TRC cluster has heterogeneity, because of their large proportion, and may include stromal cells residing in T-zone-adjacent niche such as the DCP. In agreement with this, Hara et al. generated IL-7-GFP knock-in reporter mice and observed that FRCs with high IL-7-expression are abundant in the peripheral T cell area including the CR and regions facing to the medullary sinus (44). In close contact with the DRC network, B cells localized to the DCP demonstrate migration ability that is different from that of follicular B cells, suggesting that DRCs influence B cell function. Slower and more frequent turning of B cell movement in the DCP than in the follicles might be a reflection of denser stromal obstacles and/or the absence of CXCL13 in this area. Genetic analysis using various combinations of gene deficiencies revealed that the localization of B cells in the DCP is regulated mainly by CCL21-ser and moderately by CXCL12. Interestingly, the pattern of dependency on CCL21-ser and CXCL12 is well consistent with that observed in the homing of B cells to the LNs from circulation (45). These suggest a close correlation between B cell localization in the DCP and homing into LNs through the HEVs. In addition to DRCs, CCL21-ser and CXCL12 seem to be coexpressed in pericyte-looking cells surrounding the HEVs in *Cxcl12*-EGFP/*Ccl21a*-tdTomato double reporter mice, which is also consistent with the previous report (45).

The enrichment of B cells on the medullary side might be advantageous for inducing immediate responses in the LN. Although at first lymph-borne antigens flow into the SCS, they can reach the medullary sinus at nearly the same time (46–48). It is therefore possible that some antigens are captured by DCP-B cells, and promptly stimulate these cells to produce antibodies in a T cell-independent manner. Simultaneously, the presence of DCs and T cells in the DCP might foster T cell-dependent responses *in situ*. In contrast, preferential localization of plasma cells to the MCs and close contact with the MCRC reticulum suggest that MCRCs play important roles in the construction of a supportive microenvironment for plasma cells. In fact, Huang et al. recently reported the detailed characterization of medullary FRCs and their functional aspect in plasma cell homeostasis (49). MCRCs are similar to DRCs in terms of CXCL12 expression, but high LepR expression and plasma cell localization indicate that they comprise a distinct subset. Interestingly, optimal development of the DCP and medulla is regulated by B cells. This suggests a functional link between B cells and the anatomy of the medullary side.

The present study focused on tissue structures and FSC subsets of steady-state LNs. However, as the subcompartmental structure of the LN is dramatically remodeled during immune responses (50–52), the functions and distribution of FSCs are likely to change markedly in these situations; in addition, FSCs that could be defined as a new subset with significantly different properties might be induced (37). Subcompartments specific to other secondary lymphoid organs, such as the red pulp and marginal zone of the spleen and the subepithelial dome region in Peyer's patches, might also harbor their own unique FSC subsets that are present in specific microenvironments. Such FSC diversification, specialization, and plasticity is surprising but appears to depend on dynamic interactions with a variety of mobile immune cells. This would regulate the generation of multi-layered tissue architecture in lymphoid organs for the stringent spatiotemporal regulation of immune responses.

## Ethics statement

This study was carried out in accordance with the recommendations of the Committee on Animal Research at Niigata University. The protocol was approved by the Committee on Animal Research at Niigata University.

## Author contributions

AT, MO, YaK, and TomK designed and performed the experiments. TomK wrote the manuscript. BL, MM, YT, and TN helped design the experiments, provided expertise, and edited the manuscripts. MK, IO, EU, YoK, MR, YS and TosK provided experimental help and expertise.

### Conflict of interest statement

The authors declare that the research was conducted in the absence of any commercial or financial relationships that could be construed as a potential conflict of interest.

## Supplementary material

Video S1 Differential migration of B cells in the follicle and the DCP. Related to Figure 7B. CMTMR-labeled B cells (red) were injected into a wild type B6 mouse and the explanted LN was examined by time-lapse scanning using a two-photon microscope. Frames (72-μm z-projection images) were taken every 20 s for 30 min. Playback is 10 frames/s. Collagen fibers were detected by second harmonic generation (blue). B cell migration trajectories in the follicle (F, pseudo-surfaced follicular region; trajectories, orange lines) and extrafollicular area including the deep cortex periphery and medulla (green lines) are shown.

Video S2 Dense FSC network in the DCP. Related to Figure 7G. CMTMR-labeled B cells were injected into a *Cxcl12*-EGFP mouse and the explanted LN was examined by two-photon microscopy. B cells (red) and FSCs (green) are shown. 16-μm z-projection image.

Video S3 B cell migration in the DRC network. Related to Figures 7G–I. CMTMR-labeled B cells were injected into a *Cxcl12*-EGFP mouse and the explanted LN was examined by time-lapse scanning using a two-photon microscope. Frames (16-μm z-projection images) were taken every 20 s for 20 min. Playback is 10 frames/s. B cells (red) and FSCs (green) are shown. B cell migration trajectories are shown in the right movie.

## Abbreviations

CRC: CXCL12-expressing reticular cell
DC: dendritic cell
DCP: deep cortex periphery
DRC: DCP reticular cell
FDC: follicular dendritic cell
FRC: fibroblastic reticular cell
GC: germinal center
LN: lymph node
MC: medullary cord
MCRC: medullary cord reticular cell
MRC: marginal reticular cell
MS: medullary sinus
SCS: subcapsular sinus
TRC: T zone reticular cell.
